# Hospital-Acquired Sacral Pressure Ulcer Complicated by a Spinal Epidural Abscess

**DOI:** 10.7759/cureus.60379

**Published:** 2024-05-15

**Authors:** Timothy L Carlson, Raul Alba, Qusay Alfaori, Jared J Bies, Mariela Lane, Thwe Htay

**Affiliations:** 1 Internal Medicine, Texas Tech University Health Sciences Center El Paso Paul L. Foster School of Medicine, El Paso, USA; 2 Internal Medicine, Texas Tech University Health Sciences Center El Paso, El Paso, USA

**Keywords:** treatment of spinal epidural abscess, sacral pressure ulcer, complications of bacteroides bacteremia, hospital acquired pressure injury, spinal epidural abscess

## Abstract

A spinal epidural abscess is a rare condition characterized by the accumulation of pus between the dura mater and vertebral column, often caused by hematogenous spread from a distant site or local spread from infection in nearby structures. The abscess leads to compression of the spinal cord and can result in neurological damage, including dysfunction or permanent neurological deficits. Treatment of spinal epidural abscesses should not be delayed and requires a combination of decompression by surgical drainage and antibiotic therapy. The authors present a rare case in which a spinal epidural abscess developed from a hospital-acquired pressure ulcer, further complicated by bacteremia.

## Introduction

Individuals with restricted mobility, including those undergoing hospitalization due to severe illness, are susceptible to developing pressure-induced skin and soft tissue injury, also known as pressure injuries. A pressure injury is a localized injury to the skin or underlying tissue, typically over a bony prominence, resulting from prolonged pressure with an external surface combined with shear and friction [1]. The correlation between pressure injuries as a primary cause of bacteremia has not been well studied. Most studies of bacteremia list skin and soft tissue as the source of infection and do not specifically identify pressure injuries as the source [2].

While anaerobic bacteria, including *Bacteroides fragilis*, have been isolated from chronic pressure injuries, complications such as bacteremia, spondylodiscitis, and spinal epidural abscesses arising from these cases are rare. Furthermore, these complications are most often associated with *Staphylococcus aureus* and coagulase-negative *Staphylococcus *spp. Aside from bacteremia, it is exceedingly rare for *Bacteroides* spp. to be the causative organism in spondylodiscitis and spinal epidural abscess; the English literature includes only 22 known cases of spondylodiscitis, with only 2% of those being associated with spinal epidural abscess [2-8].

We report a case in which a hospital-acquired pressure injury led to *Bacteroides*-associated bacteremia, spondylodiscitis, and a large spinal epidural abscess in a 41-year-old male.

## Case presentation

A 41-year-old male with a past medical history of non-insulin-dependent type 2 diabetes mellitus, hypertension, and alcohol use disorder presented to the emergency department with altered mental status and a draining, foul-smelling bed sore over his sacrum. History revealed that four days prior, he was discharged from another facility after a 12-day hospitalization for treatment of alcohol withdrawal, hyponatremia, and urinary retention, during which he developed a sacral pressure injury. Additionally, at the time of discharge, he was provided with a seven-day prescription for trimethoprim-sulfamethoxazole but was unable to fill the prescription. The patient’s mental status subsequently deteriorated and family brought him to our facility to be evaluated and treated.

On presentation to the emergency department, the patient had a temperature of 39.5°C, heart rate of 102 beats per minute, respiratory rate of 17 breaths per minute, blood pressure of 121/62 mmHg, and oxygen saturation of 98% on room air. He complained of photophobia, weakness, dysuria, and diarrhea. The family at the bedside reported that these symptoms had started days after his previous hospital discharge and progressively worsened, with the patient becoming increasingly confused and unable to ambulate due to weakness.

On physical examination, the patient appeared somnolent and disoriented to time and place, but showed no signs of meningismus, nuchal rigidity, or papilledema, with intact pupillary light reflexes. Motor strength in the bilateral lower extremities was diminished to 2/5, with absent plantar reflexes and bilateral loss of sensation to light touch below the T8 level. The abdominal exam revealed distension in the lower abdominal region and tenderness upon palpation in the suprapubic area.

The patient was started empirically on vancomycin (2000 mg IV q12hrs), ceftriaxone (2g IV q12hrs), ampicillin (2g IV q4hrs), and acyclovir (700mg IV q8hrs), as well as dexamethasone (10mg IV q6hrs). Neurology, neurosurgery, and general surgery were consulted due to high clinical concerns of an epidural abscess causing cauda equina syndrome symptoms. Initial laboratory results showed leukocytosis with neutrophil predominance, normocytic anemia, mild hyponatremia, hypokalemia, hypoalbuminemia, transaminitis, prolonged PT/PT-INR, and elevated acute phase reactants (Table 1). Blood and urine cultures were ordered.

**Table 1:** Initial Laboratory Work-Up INR: International Normalized Ratio.

Variable	Patient Result	Reference Range
White blood cells	12.95 × 10^3^/µL	4.50 × 10^3^ to 11.00 × 10^3^ mL^-1^
Neutrophils	10.47 × 10^3^/µL	2.00 × 10^3^ to 7.8 × 10^3^ mL^-1^
Lymphocytes	1.51 × 10^3^/µL	1.00 × 10^3^ to 4.8 × 10^3^ mL^-1^
Monocytes	0.77 × 10^3^/µL	0.10 × 10^3^ to 1.00 × 10^3^ mL^-1^
Eosinophils	0.09 × 10^3^/µL	0.00 × 10^3^ to 0.70 × 10^3^ mL^-1^
Basophils	0.05 × 10^3^/µL	0.00 × 10^3^ to 0.20 × 10^3^ mL^-1^
Hemoglobin	8.3 G/dL	12.0–15.0 G/dL
Hematocrit	25%	36%–47%
Mean corpuscular volume	90.6 fL	82–98 fL
Platelets	399 × 10^3^/µL	150 × 10^3^ to 450 × 10^3^ mL^-1^
Sodium	131 mmol/L	135–145 mmol/L
Potassium	3.2 mmol/L	3.5–5.1 mmol/L
Chloride	96 mmol/L	98–107 mmol/L
Anion gap	15 mmol/L	5–19
Glucose	151 mg/dL	74–106 mg/dL
Blood urea nitrogen	13 mg/dL	7–17 mg/dL
Creatinine	0.5 mg/dL	0.52–1.04 mg/dL
Calcium	8.5 mg/dL	8.4–10.2 mg/dL
Albumin	2.9 G/dL	3.5–5.0 g/dL
Protein	7.2 G/dL	6.3–8.2 g/dL
Total bilirubin	0.7 mg/dL	0.2–1.3 mg/dL
Albumin/globulin ratio	0.7	1.4–1.6
Aspartate transaminase	77 Intern Unit/L	14–46 U/L
Alkaline phosphatase	121 Intern Unit/L	38–126 U/L
Alanine transaminase	113 Intern Unit/L	0–35 U/L
Prothrombin time	17.8 seconds	11.8–14.8 seconds
Prothombin time - INR	1.5 seconds	0.9–1.1 seconds
Activated partial thomboplastin time	34.5 seconds	23.3–38.6 seconds
Erythrocyte sedimentation rate	133 mm/hour	0–14 mm/hour
C-reactive protein	11.72 mg/dL	0–1 mg/dL

A head CT showed no acute intracranial abnormality. A CT of the lumbar spine showed irregular endplates at L5-S1 with minimal surrounding soft tissue, along with the presence of gas within the spinal canal and L5-S1 disc space. A lumbar puncture performed in the emergency department yielded 1 mL of purulent fluid. Cerebrospinal fluid (CSF) analysis showed pale yellow purulent fluid, elevated white blood cell count with neutrophil predominance, hypoglycorrhachia, and a gram stain positive for gram-negative bacilli (Table 2). These results led the primary team to believe that the CSF was inadequately sampled and that the lumbar puncture likely yielded fluid from an epidural abscess.

**Table 2:** CSF Results on Admission CSF: cerebrospinal fluid.

Variable	Patient Result	Reference Range
Volume (mL)	0.75 mL	Variable
Color	Pale yellow	Clear
Appearance	Turbid	Clear
Total nucleated cells (cells/mm^3^ or cells/µL)	798,600	0–5
Red blood cells (cells/mm^3^ or cells/µL)	91,000	Nil
Segmented neutrophil %	50	0
Lymphocyte %	25	70
Macrophage %	25	30
Glucose (mg/dL)	26	50-80
Gram stain	Many gram-negative bacilli	No microorganism

**Figure 1:**
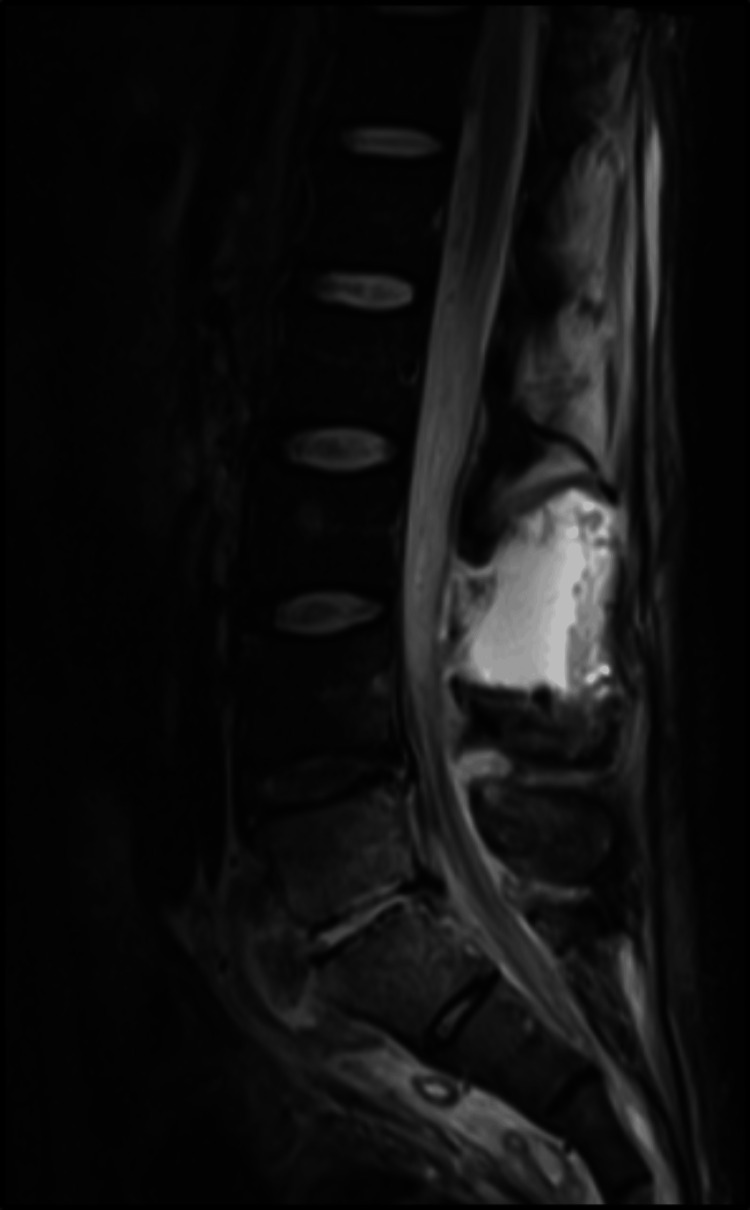
Sagittal view showing enhanced epidural abscess.

**Figure 2:**
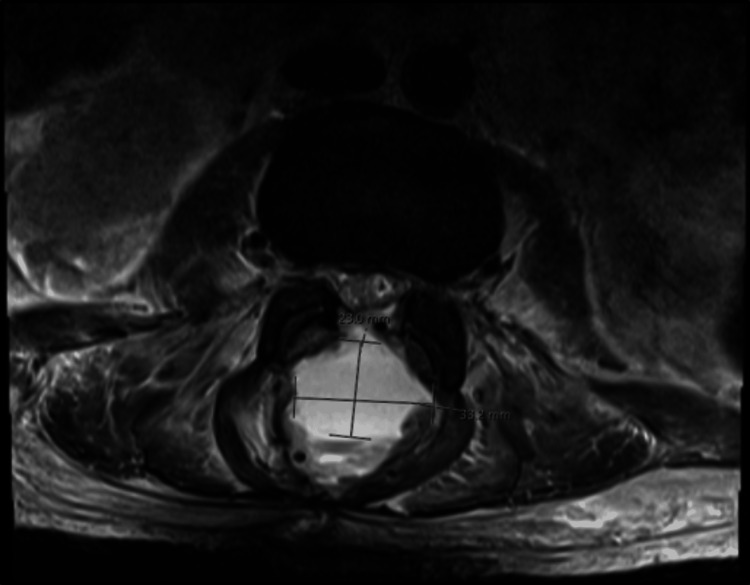
Transverse view showing enhanced 33 mm x 23 mm epidural abscess.

**Figure 3:**
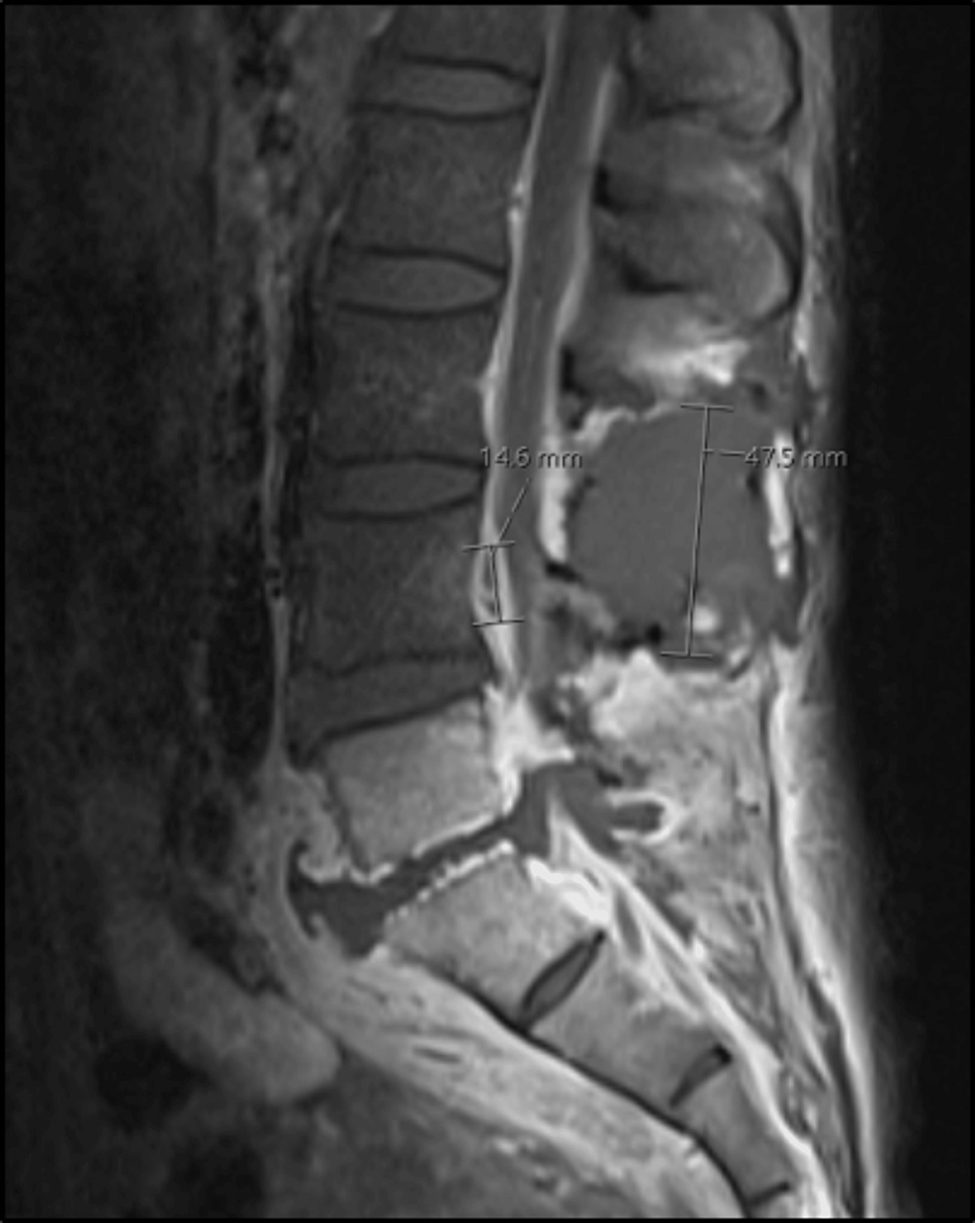
Sagittal view showing 47.5 mm and 14.6 mm epidural abscess.

**Figure 4:**
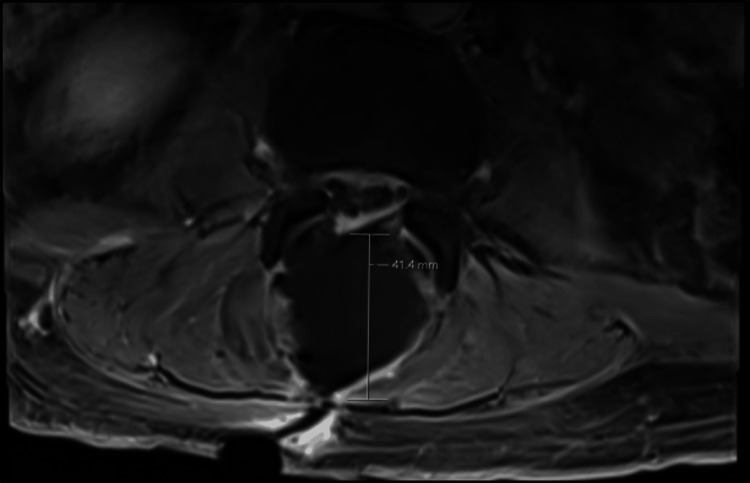
Transverse view showing 41 mm epidural abscess.

MRI of the lumbar spine was significant for spondylodiscitis from L5-S1 with associated significant regional soft tissue swelling, multiple soft tissue abscesses, and an extensive epidural abscess with superimposed spondylosis. This resulted in moderate spinal canal stenosis from L1-L2 through L3-L4, severe spinal canal stenosis at L4-L5 and L5-S1, and severe foraminal narrowing at L5-S1 (Figures 1-4). MRI of the head, cervical, and thoracic spine were unremarkable.

Infectious disease was consulted, and ceftriaxone, acyclovir, and ampicillin were discontinued due to the low likelihood of meningitis. Vancomycin was continued intravenously, and cefepime 2 g IV q8hrs was added, with the duration planned to be determined after neurosurgical intervention and evaluation of intraoperative cultures. Neurosurgery and general surgery performed surgical intervention, including epidural phlegmon evacuation, L3-L4 laminectomy, and ulcer debridement. Post-operation, a JP drain was secured, and he was subsequently admitted to the surgical intensive care unit.

Several *Bacteroides* species, including *Bacteroides fragilis*, *Bacteroides pneumosintes*, and *Bacteroides thetaiotaomicron*, were isolated from the abscess, wound cultures, and blood cultures. Infectious disease recommended adjusting the antibiotic treatment to cefepime and metronidazole, with a planned treatment duration of at least six weeks.

The patient improved clinically and was discharged on hospital day 13 with oral metronidazole and levofloxacin to be continued in the outpatient setting. Four days later, the patient was readmitted with sepsis, failure of the wound VAC, and a concern of abscess at the laminectomy site, which was managed medically with fluid resuscitation, wound care, and IV cefepime and metronidazole. After stabilization, he was discharged on hospital day 6 with a plan for a continued course of oral levofloxacin and metronidazole.

A subsequent MRI of the lumbar spine two months after surgery revealed improving spondylodiscitis but persistent extensive regional soft tissue thickening and inflammation with superimposed lumbar spine spondylosis. Infectious disease recommended continued antibiotic treatment for 12 weeks. At his four-month follow-up appointment, he was noted to be doing very well. He had regained strength in both lower extremities and was walking without assistance.

## Discussion

A spinal epidural abscess is a collection of pus lying between the dura mater and the overlying vertebral column. It is a rare diagnosis, occurring in about 2/10,000 hospital patients, and can occur when bacteria gain access to the epidural space by hematogenous dissemination from elsewhere in the body, or less commonly, through contiguous spread from a local infection such as a pressure ulcer [3, 7, 9-11]. It is rare for a hospital-acquired pressure injury to develop into an epidural abscess [3, 6].

Hematogenous spread occurs in 25 to 50% of patients and is often secondary to skin infections, urinary tract infections, pneumonia, or pharyngitis [4]. Localized spread occurs in about one-third of cases due to sources including pressure ulcers, vertebral osteomyelitis, paraspinal abscess, infection after penetrating trauma, back surgery, lumbar puncture, CT-guided needle biopsies, and administration of epidural anesthesia or analgesia [3, 4, 7, 12].

Diabetes mellitus is an important risk factor and has been implicated in 50% of patients with spinal epidural abscess [3, 9-12]. Other important predisposing factors include medical conditions such as bacteremia, alcohol use disorder, HIV infection, malignancy, and renal insufficiency.

Most risk factors facilitate the spread of infection by skin flora, with two-thirds of cases implicating *Staphylococcus aureus *as the causative organism. Less common pathogens include *Staphylococcus epidermidis*, *Escherichia coli*, and *Pseudomonas *spp. [4, 7, 13-15]. Rare causes include anaerobic bacterial infection, actinomycosis, nocardiosis, tuberculous and non-tuberculous mycobacteria, fungi including *Candida* spp., *Sporothrix *spp., and *Aspergillus *spp., and parasites including *Echinococcus *spp. and *Dracunculus *spp. [4, 13-15].

Spinal epidural abscesses can have considerable variability in clinical presentation, severity, and progression of neurological symptoms [16]. The classic triad of spinal pain, fever, and neurological deficits is not reported in every case [16]. Severe localized back pain is the most commonly reported symptom, and when present, it should alert the clinician that further diagnostic testing is needed [5, 9]. When spinal epidural abscess is suspected, frequent neurological exams are necessary [5, 9].

When considering a spinal epidural abscess diagnosis, C-reactive protein levels will almost always be elevated; however, C-reactive protein lacks specificity [11]. Magnetic resonance imaging serves as the gold standard for diagnosis, exceeding 90% sensitivity and specificity, and should be prioritized when a spinal epidural abscess is suspected [17]. While blood cultures can offer insight into antibiotic therapy, studies suggest they will return negative results in 40% of cases [16]. As part of a comprehensive approach, microbiological studies should be done to identify potential sources of infection.

When addressing management, adopting a multidisciplinary approach is crucial, with an emphasis on thorough evaluation. For empiric antimicrobial treatment, coverage for *Staphylococcus* spp., *Streptococci*, and gram-negative bacteria is recommended [9]. Surgical decompression and drainage remain the treatment of choice to minimize the risk of neurological damage [5]. Typically, the duration of antimicrobial treatment for spinal epidural abscess is 4-6 weeks, extending to 8-12 weeks in cases involving concomitant vertebral osteomyelitis [5, 11].

When considering the final neurological outcome, there is a strong correlation with the severity and duration of neurological deficits preceding surgery [5, 9]. A persistently elevated C-reactive protein level is a prognostic factor when monitoring the disease course. It is important to acknowledge that paralysis persisting beyond 24-36 hours is unlikely to show improvement [5, 11].

## Conclusions

This report presents the unique case of a patient who developed a spinal epidural abscess and bacteremia secondary to the hematogenous spread of *Bacteroides* spp. from a hospital-acquired stage 4 sacral pressure ulcer. Although most cases of spinal epidural abscess involve Staphylococcal or Streptococcal species, current literature identifies rare cases involving *Bacteroides* spp. However, the literature is lacking in cases of spinal epidural abscess, spondylodiscitis, and bacteremia as sequelae of hospital-acquired pressure injury, particularly involving the rare causative organism *Bacteroides* spp.

Spinal epidural abscess is a grave condition that requires prompt recognition and treatment to avoid permanent neurological damage. Clinicians must have a high index of suspicion even when patients do not present with the classic triad of back pain, fever, and neurological deficits. Congruent with our case, the presence of diabetes and alcohol use disorder are facets of the patient history that should raise suspicion for this diagnosis. Furthermore, derangements of PT/INR/PTT can offer some insight into Bacteroides bacteremia, particularly when acquiring a lumbar puncture is challenging.
